# Polarization-Multiplexed Holography Based on Dielectric Metasurfaces

**DOI:** 10.3390/mi17101179

**Published:** 2026-10-11

**Authors:** Chen He, Xizhi Wan, Jingyao Hou, Ziyu Tian, Wang Wang, Nian Kang, Shun Zhou, Xueping Sun

**Affiliations:** 1 School of Opto-Electronical Engineering, Xi’an Technological University, Xi’an 710021, China; neckerchen@163.com (C.H.);; 2 Xi’an North Electro-Optic Science & Technology Defense Co., Ltd., Xi’an 710043, China

**Keywords:** all-dielectric, metasurface, phase modulation, polarization multiplexing, holographic imaging

## Abstract

We present a silicon-nanobrick metasurface for dual-channel polarization-multiplexed holography at 1.55 μm. A Jones-matrix design combined with a Fresnel-based Gerchberg–Saxton algorithm maps the target phase profiles to nanobrick dimensions and orientations, enabling combined propagation-phase and geometric-phase control. Numerical simulations demonstrate holographic reconstruction for orthogonal linear, circular, and elliptical polarization pairs. The selected meta-atoms exhibit simulated power transmittances above 70% along both principal axes under periodic boundary conditions. The circular-polarization design is fabricated using electron beam lithography and hard-mask etching, and polarization-selective far-field reconstruction is experimentally observed. Further numerical analysis demonstrates continuous variation of the relative reconstructed intensities along an incident-polarization trajectory. Under an ideal collection and analyzer model, separately optimized elliptical analyzers provide central-signal suppression of 19.02 and 18.78 dB while retaining 64.30% and 61.71% of the corresponding target-image signals. An additional simulated design reconstructs two images within the same spatial region, yielding polarization-channel extinction ratios of 9.30 and 9.33 dB under fixed circular-analyzer conditions. These results demonstrate a silicon-based implementation of dual-channel holographic encoding and provide quantitative characterization of polarization-selective readout.

## 1. Introduction

Metamaterials are artificially engineered materials characterized by unique structural designs and material compositions. Their subwavelength structural design enables tailored electromagnetic responses. Early theoretical work examined media with simultaneously negative permittivity and permeability [1], while engineered conducting structures provided a route to artificial magnetic responses [2]. Negative refractive indices were subsequently demonstrated in microwave and optical metamaterials [3,4,5] and in chiral metamaterials [6]. However, the three-dimensional architectures of conventional metamaterials introduce substantial fabrication challenges and costs. Additionally, early metamaterials predominantly composed of metallic components suffer from severe energy dissipation due to Ohmic losses, which have hindered their widespread development and practical applications. Recent advancements in metamaterial research have revealed that two-dimensional configurations—single- or few-layer structures—exhibit remarkable electromagnetic wave manipulation capabilities. These 2D analogues, termed metasurfaces, offer advantages including simplified fabrication processes, ease of integration, and reduced material consumption. Metallic metasurfaces have demonstrated wavefront control through spatially varying phase responses, including conversion between propagating and surface waves [7] and the realization of planar lenses and axicons at telecommunication wavelengths [8]. Ohmic losses in metallic elements can limit optical efficiency, motivating the development of dielectric metasurfaces. Dielectric materials such as Si and TiO2 offer low optical absorption within suitable operating bands and enable phase modulation through the dimensions and orientations of their meta-atoms. Metasurfaces have been applied to holographic displays [9,10,11], metalenses [12,13,14], and beam transformations [15,16,17]. In holography, metasurfaces exhibit exceptional advantages due to their customizable wavefront control, multifunctionality, compact footprint, high efficiency, and design flexibility. By tailoring the geometry, dimensions, and spatial arrangement of subwavelength meta-atoms, precise manipulation of optical parameters—amplitude [18,19], phase [20,21,22], complex amplitude [23,24,25], and polarization [26,27,28]—can be achieved. Compared to conventional holographic plates, metasurface-based holograms offer simplified encoding methodologies and larger information capacity, enabling high-fidelity image reconstruction.

Polarization-multiplexed metasurface holography has progressed toward increasingly flexible control of optical channels. Li et al. developed a framework that accommodates variable polarization-conversion efficiencies, enabling separate control of channel phases and energy allocation, with experimental demonstrations involving three polarization channels [29]. Hu et al. demonstrated a noninterleaved TiO_2_ metasurface combining three polarization channels with trichromatic holographic reconstruction [30]. These studies establish representative approaches to polarization-dependent wavefront control and multichannel information encoding.

Here, we investigate dual-channel holographic reconstruction using silicon nanobricks on a SiO_2_ substrate at 1.55 μm. A Jones-matrix-based design procedure maps the target phase profiles to nanobrick dimensions and orientations, supporting designs for orthogonal linear, circular, and elliptical polarization pairs. The circular-polarization design is fabricated and experimentally characterized. The study further quantifies the reconstructed intensity along an incident-polarization trajectory and evaluates polarization filtering for central-spot suppression. An additional design reconstructs two images within the same spatial region, yielding channel extinction ratios of 9.30 and 9.33 dB under fixed ideal circular-analyzer conditions. Together, these results connect device design and experimental implementation with quantitative characterization of polarization-selective holographic readout.

## 2. Theory of Orthogonally Polarization-Multiplexed Holography

When electromagnetic waves are incident on a metasurface composed of anisotropic materials, the following relationships hold:

(1) $$\left[\begin{array}{c} E_{\mathrm{xout}}\\ E_{\mathrm{yout}} \end{array}\right]=J\left[\begin{array}{c} E_{\mathrm{xin}}\\ E_{\mathrm{yin}} \end{array}\right]$$

where *E_xout_* and *E_yout_* denote the polarization components along the x and y directions of the emergent electromagnetic wave, respectively; *E_xin_* and *E_yin_* represent the polarization components along the x and y directions of the incident electromagnetic wave; and J is the Jones matrix of the anisotropic metasurface, expressed as:

(2) $$J=\left[\begin{array}{cc} J_{11} & J_{12}\\ J_{21} & J_{22} \end{array}\right]$$

Assuming that the anisotropic structure corresponds to the u-v coordinate system while the entire metasurface corresponds to the x-y coordinate system, the angle between the two coordinate systems is denoted as *θ*. At this point, the Jones matrix of the anisotropic metasurface can be expressed as:

(3) $$J=R(-\theta )\left[\begin{array}{cc} t_{u} & 0\\ 0 & t_{v} \end{array}\right]R(\theta )$$

where the complex amplitudes of the anisotropic structures along their major and minor axes are denoted as *t_u_* and *t_v_*, respectively. The rotation matrix *R*(θ) can be expressed as:

(4) $$R(\theta )=\left[\begin{array}{cc} \cos\theta & \sin\theta \\ -\sin\theta & \cos\theta \end{array}\right]$$

By substituting Equation (4) into Equation (3) and simplifying, we obtain:

(5) $$J=\left(\begin{array}{cc} t_{u}\cos^{2}\theta +t_{v}\sin^{2}\theta & (t_{u}-t_{v})\sin\theta \cos\theta \\ (t_{u}-t_{v})\sin\theta \cos\theta & t_{u}\sin^{2}\theta +t_{v}\cos^{2}\theta \end{array}\right)$$

For the derivation of the phase-design relations, a normalized phase-only approximation is adopted, with the principal-axis transmission amplitudes set to unity. The corresponding transmission coefficients are expressed as $t_{u}=e^{i\varphi_{x}}$ and $t_{v}=e^{i\varphi_{y}}$, where *φ_x_* and *φ_y_* denote the phase responses along the two principal axes, respectively. Under this approximation, Equation (5) can be rewritten as:

(6) $$J=\left[\begin{array}{cc} e^{i\varphi_{x}}\cos^{2}\theta +e^{i\varphi_{y}}\sin^{2}\theta & (e^{i\varphi_{x}}-e^{i\varphi_{y}})\sin\theta \cos\theta \\ (e^{i\varphi_{x}}-e^{i\varphi_{y}})\sin\theta \cos\theta & e^{i\varphi_{x}}\sin^{2}\theta +e^{i\varphi_{y}}\cos^{2}\theta \end{array}\right]$$

Equation (6) gives the normalized phase-only Jones matrix used to derive the polarization-multiplexing design relations. The actual amplitude and phase responses of the nanobricks are included in the subsequent FDTD simulations. Next, consider the incidence of a pair of arbitrary orthogonal polarization states:

(7) $$\begin{array}{l} \lambda^{+}={\left[\begin{array}{cc} \lambda_{1}^{+} & \lambda_{2}^{+} \end{array}\right]}^{T}\\ \lambda^{-}={\left[\begin{array}{cc} \lambda_{1}^{-} & \lambda_{2}^{-} \end{array}\right]}^{T} \end{array}$$

The output polarization state is:

(8) $$\begin{array}{l} \kappa^{+}={\left[\begin{array}{cc} \kappa_{1}^{+} & \kappa_{2}^{+} \end{array}\right]}^{T}\\ \kappa^{-}={\left[\begin{array}{cc} \kappa_{1}^{-} & \kappa_{2}^{-} \end{array}\right]}^{T} \end{array}$$

In the equation, the superscript T denotes the matrix transpose, where $\kappa^{\pm }={\left(\lambda^{\pm }\right)}^{*}$, and ∗ represents the complex conjugate, *φ*^±^ corresponds to any pair of phase profiles. Consequently, the Jones matrix of the device must satisfy:

(9) $$\begin{array}{l} J\lambda^{+}=e^{i\varphi^{+}}\kappa^{+}\\ J\lambda^{-}=e^{i\varphi^{-}}\kappa^{-} \end{array}$$

The simplification results are as follows:

(10) $$J=\left[\begin{array}{cc} e^{i\varphi^{+}}{\left(\lambda_{1}^{+}\right)}^{*} & e^{i\varphi^{-}}{\left(\lambda_{1}^{-}\right)}^{*}\\ e^{i\varphi^{+}}{\left(\lambda_{2}^{+}\right)}^{*} & e^{i\varphi^{-}}{\left(\lambda_{2}^{-}\right)}^{*} \end{array}\right]{\left[\begin{array}{cc} \lambda_{1}^{+} & \lambda_{1}^{-}\\ \lambda_{2}^{+} & \lambda_{2}^{-} \end{array}\right]}^{-1}$$

**Figure 1:**
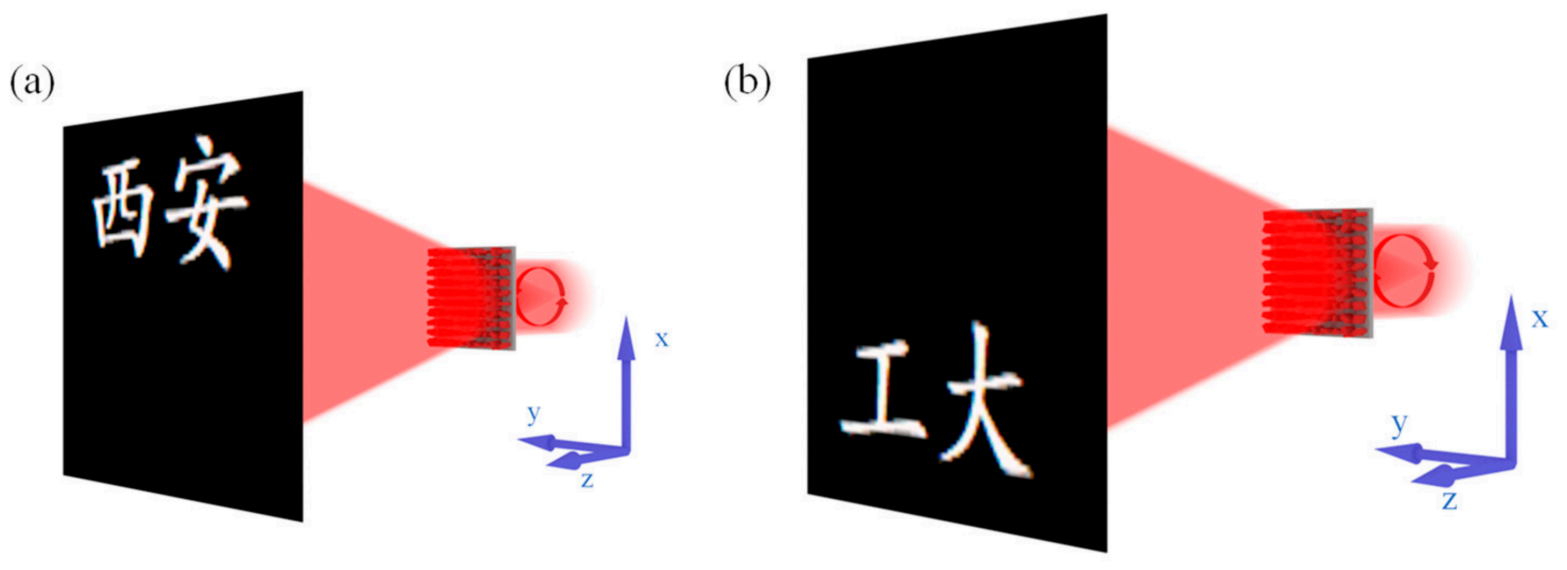
Schematic diagram of orthogonal polarization-multiplexed holographic display (**a**) LCP (**b**) RCP. The Chinese characters in panels (**a**) and (**b**) read “Xi’an” (the name of a city in China) and “Gongda” (an abbreviation of *Gongye Daxue*, meaning “University of Technology”), respectively.

By equating Equation (10) with Equation (6) and substituting the orthogonal linear polarizations ($\lambda^{+}={\left[\begin{array}{cc} 1 & 0 \end{array}\right]}^{T}$ and $\lambda^{-}={\left[\begin{array}{cc} 0 & 1 \end{array}\right]}^{T}$), orthogonal circular polarizations ($\lambda^{+}={\left[\begin{array}{cc} 1 & i \end{array}\right]}^{T}/\sqrt{2}$ and $\lambda^{-}={\left[\begin{array}{cc} 1 & -i \end{array}\right]}^{T}/\sqrt{2}$), and orthogonal elliptical polarizations ($\lambda^{+}={\left[\begin{array}{cc} 1 & 2i \end{array}\right]}^{T}/\sqrt{5}$ and $\lambda^{-}=2{\left[\begin{array}{cc} 1 & -0.5i \end{array}\right]}^{T}/\sqrt{5}$) respectively, we solve for parameters *φ_x_*, *φ_y_*, and *θ*. The calculation results are shown in Equations (11)–(13). In the subsequent arrangement of unit cell arrays, appropriately sized unit structures are selected based on parameters *φ_x_* and *φ_y_*, then assigned the corresponding rotation angle *θ*. This completes the design of the polarization-multiplexed holographic metasurface device, which enables target image reconstruction through numerical simulations. Figure 1 shows the imaging principle using orthogonal circular polarization-multiplexed holography as an example.

*φ_x_*(*x*,*y*) = *φ*^+^(*x*,*y*),  *φ_y_*(*x*,*y*) = *φ*^−^(*x*,*y*),  *θ* = 0 (11)

(12) $$\begin{array}{l} \varphi_{x}(x,y)=\frac{\varphi^{+}(x,y)+\varphi^{-}(x,y)}{2}\\ \varphi_{y}(x,y)=\frac{\varphi^{+}(x,y)+\varphi^{-}(x,y)}{2}-\pi \\ \theta (x,y)=\frac{\varphi^{+}(x,y)-\varphi^{-}(x,y)}{4} \end{array}$$

(13) $$\begin{array}{l} \varphi_{x}(x,y)=-i\ln\frac{e^{i\varphi^{+}(x,y)}+4e^{i\varphi^{-}(x,y)}}{5(\cos^{2}\theta (x,y)+\sin^{2}\theta (x,y)e^{\mathrm{ia}(x,y)})}\\ \varphi_{y}(x,y)=\varphi_{x}(x,y)+a(x,y)\\ a(x,y)=2\arctan(-\frac{4}{3\sin2\theta (x,y)})\\ \theta (x,y)=\frac{1}{2}\arctan(\frac{4}{5}\tan\frac{\varphi^{+}(x,y)-\varphi^{-}(x,y)}{2}) \end{array}$$

## 3. Simulation and Results

### 3.1. Design of Unit Cells

**Figure 2:**
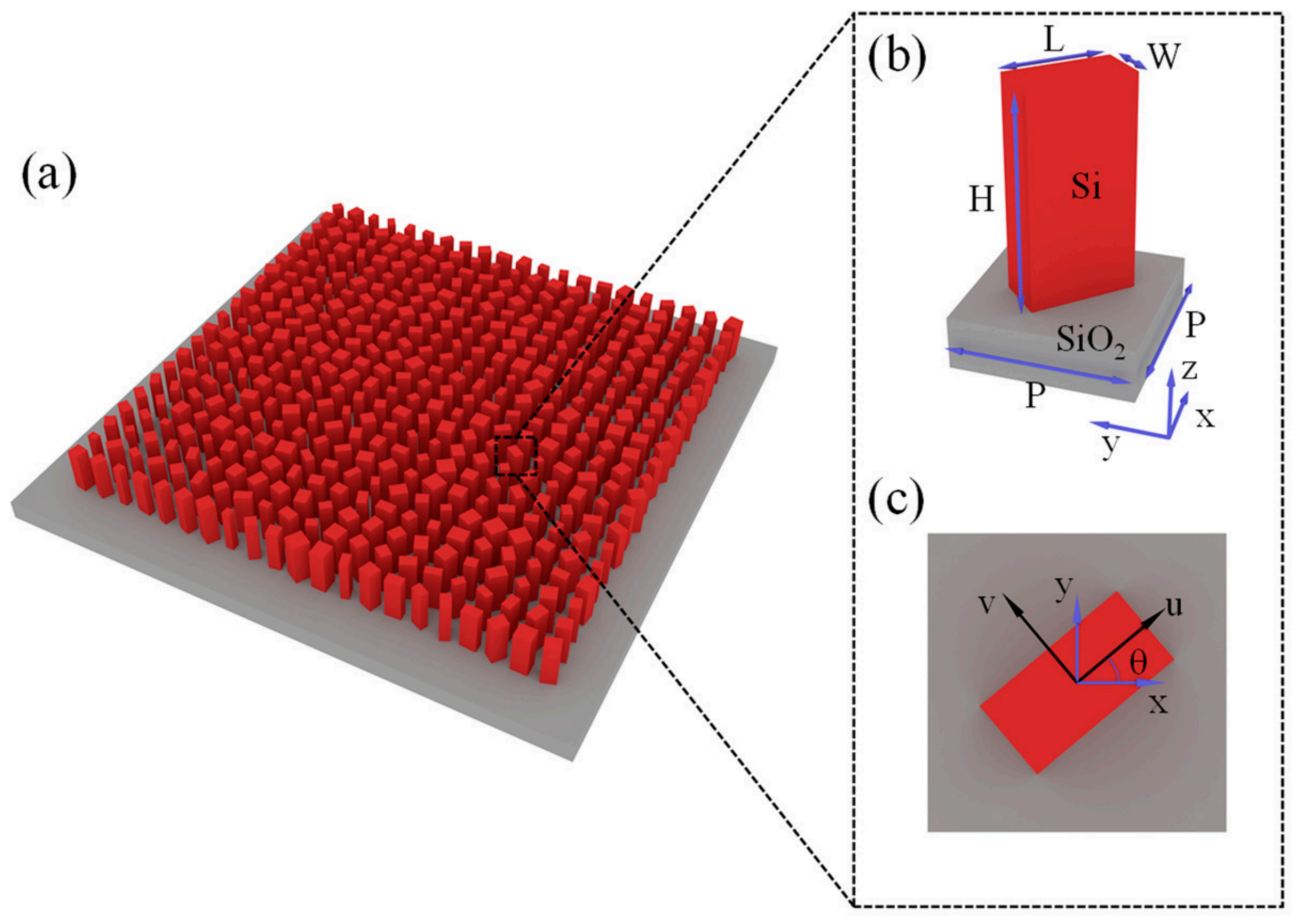
Schematic diagram of the metasurface hologram: (**a**) The metasurface based on a basic nanobrick structure; (**b**) The unit cell diagram in one period; (**c**) The top view of the unit cell.

For the operational wavelength of 1.55 μm, Si is selected as the material for phase-tuning elements. With a refractive index of approximately 3.5 and a near-zero extinction coefficient, silicon satisfies the requirements of high refractive index and low absorption. The substrate material employs SiO_2_, which exhibits a refractive index of 1.5 and a negligible extinction coefficient at the wavelength of 1.55 μm, meeting the criteria of low refractive index and high transmittance. Both Si and SiO_2_ demonstrate compatibility with standard semiconductor fabrication processes, facilitating subsequent device manufacturing. The unit structure features a rectangular cross-section for enhanced fabrication feasibility, as shown in Figure 2. In the schematic, the Si nanobrick is characterized by height H, length L, width W, and rotation angle θ, with periodicity P of the substrate array. The substrate thickness exhibits negligible influence on device performance. Through strategic arrangement of unit structures with controlled dimensional parameters and rotation angles, the resulting metasurface enables independent phase modulation for orthogonal polarization states, thereby achieving polarization-multiplexed holographic functionality.

**Figure 3:**
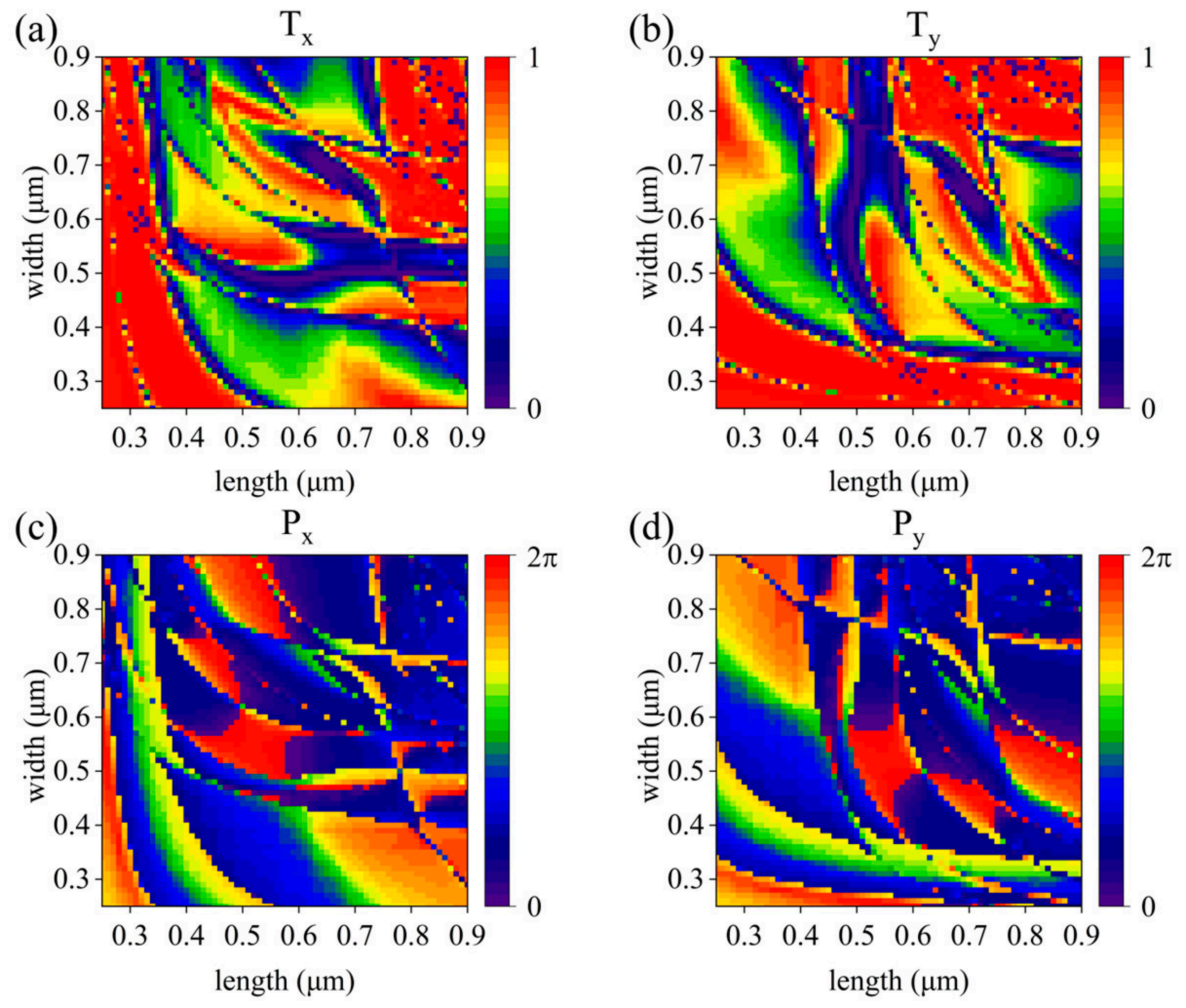
The scanning results of the unit structure with a period P = 1 μm and height H = 1.2 μm: (**a**) transmittance in the x-direction (**b**) transmittance in the y-direction (**c**) phase modulation in the x-direction and (**d**) phase modulation in the y-direction.

Simulations were conducted using the commercial finite-difference time-domain software FDTD Solutions (2020R2) to optimize the geometric parameters of the unit cell structure. A structural model was established in FDTD with periodic boundary conditions applied along the x- and y-directions, while perfectly matched layer (PML) boundary conditions were implemented in the z-direction to truncate optical waves at boundaries and eliminate effects caused by scattering and reflection. A linearly polarized light in the x-direction was normally incident from the substrate. To ensure that the structural phase values covered the full 0–2π range across varying dimensions while maintaining a low aspect ratio, iterative parameter adjustments were performed. The optimized parameters were determined as period P = 1 μm and height H = 1.2 μm, with both the nanobrick length L and width W varying from 0.25 to 0.9 μm in 0.01 μm increments. This configuration enabled comprehensive characterization of both transmittance and phase modulation for transmitted light. The maximum aspect ratio of the structure was 4.8:1, meeting fabrication requirements. Figure 3 shows the x-direction transmittance *T_x_*, phase modulation *P_x_*, y-direction transmittance *T_y_* and phase modulation *P_y_*. Parameters *T_y_* and *P_y_* were derived by transposing the distribution matrices of *T_x_* and *P_x_*. As shown in Figure 3c, the phase modulation range fully covers 0–2π, satisfying the requirements for complete phase control.

### 3.2. Fresnel Diffraction-Based Gerchberg-Saxton Algorithm

**Figure 4:**
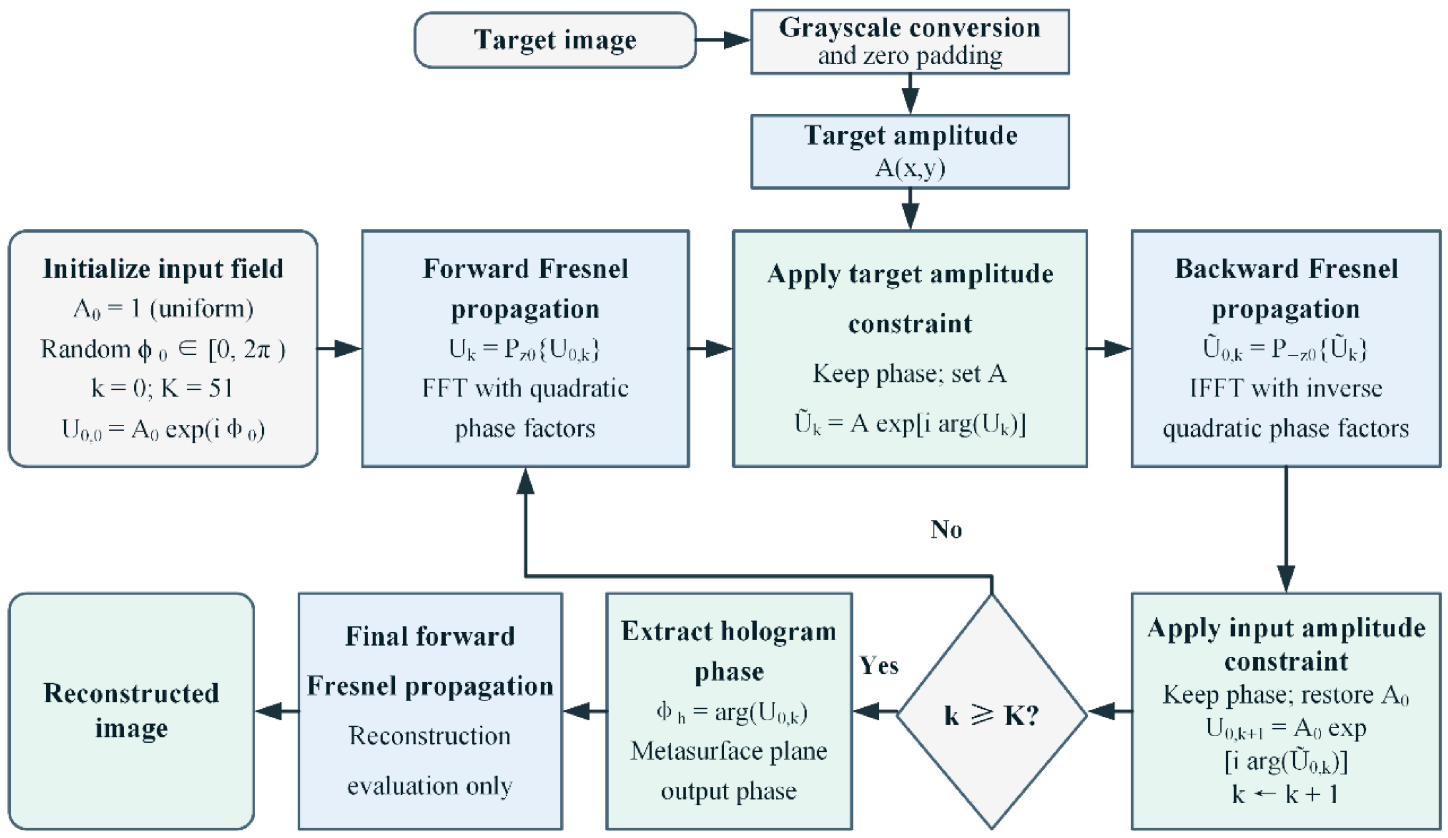
Flowchart of the Fresnel-based GS algorithm.

The phase-only holograms were generated using a Gerchberg–Saxton (GS) algorithm based on Fresnel propagation, as illustrated in Figure 4. Forward and backward propagation were implemented using FFT and IFFT operations combined with the corresponding quadratic phase factors. The design procedure is described below.

(1) Convert the target image to grayscale and embed it in a zero-padded *N* × *N* array. The grayscale values are used as the target amplitude constraint *A*(*x*,*y*) in the reconstruction plane.
(2) Set a uniform incident amplitude *A*_0_(*x*_0_,*y*_0_) = 1 and generate an initial random phase *ϕ*_0_(*x*_0_,*y*_0_) distributed between 0 and 2*π*. The initial hologram-plane field is constructed as *U*_0,0_(*x*_0_,*y*_0_) = *A*_0_(*x*_0_,*y*_0_)exp[*iϕ*_0_(*x*_0_,*y*_0_)].
(3) Propagate the hologram-plane field to the reconstruction plane. The field is first multiplied by the input-plane Fresnel quadratic phase factor, followed by an FFT and the output-plane propagation factor, yielding the reconstructed field *U_k_*(*x*,*y*).
(4) Preserve the phase of *U_k_*(*x*,*y*) and replace its amplitude with the target amplitude constraint: ${\tilde{U}}_{k}(x,y)=A(x,y)\exp[i\arg U_{k}(x,y)]$.
(5) Back-propagate ${\tilde{U}}_{k}(x,y)$ to the hologram plane using an IFFT combined with the inverse Fresnel quadratic phase factors, obtaining the field ${\tilde{U}}_{0,k}(x_{0},y_{0})$.
(6) Preserve the phase of the back-propagated field and restore the uniform incident amplitude: $U_{0,k+1}(x_{0},y_{0})=A_{0}(x_{0},y_{0})\exp\left[i\arg{\tilde{U}}_{0,k}(x_{0},y_{0})\right]$
(7) Repeat Steps 3–6 until the prescribed number of iterations is reached. The phase of the final hologram-plane field is extracted as the phase-only hologram, and a final forward propagation is performed to evaluate the reconstructed image.

**Figure 5:**
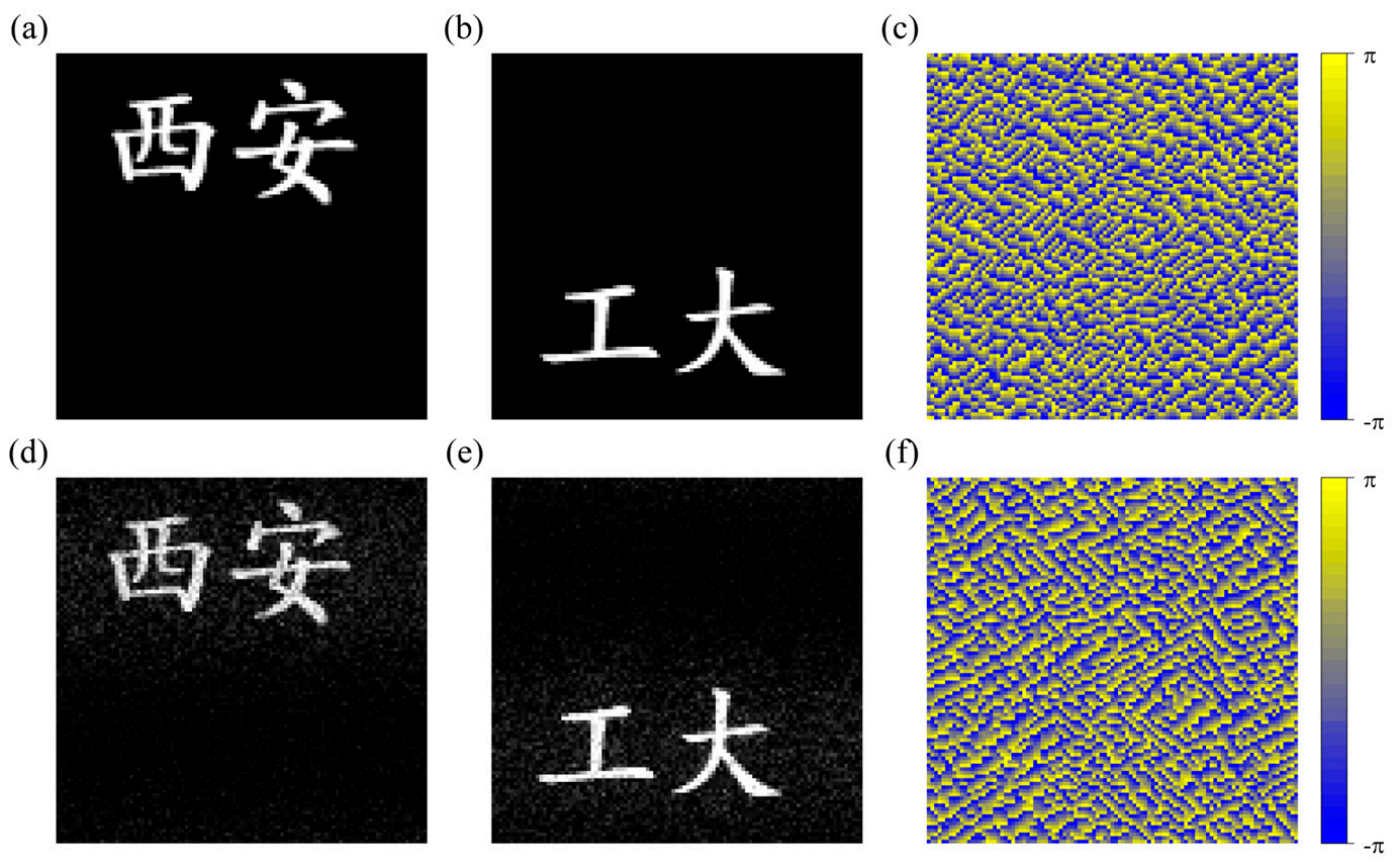
(**a**,**b**) Original image, (**d**,**e**) reconstructed image, (**c**,**f**) phase distribution. The Chinese characters in panels (**a**) and (**b**) read “Xi’an” (the name of a city in China) and “Gongda” (an abbreviation of *Gongye Daxue*, meaning “University of Technology”), respectively.

The phase information of the target images shown in Figure 5a,b was reconstructed using the Fresnel diffraction-based GS algorithm. The reconstructed images are presented in Figure 5d,e, while the corresponding hologram phase distributions are displayed in Figure 5c,f. As evident from the results, the characters in the target images can be accurately reconstructed with high precision.

### 3.3. Orthogonal Polarization Multiplexed Holographic Simulation

**Figure 6:**
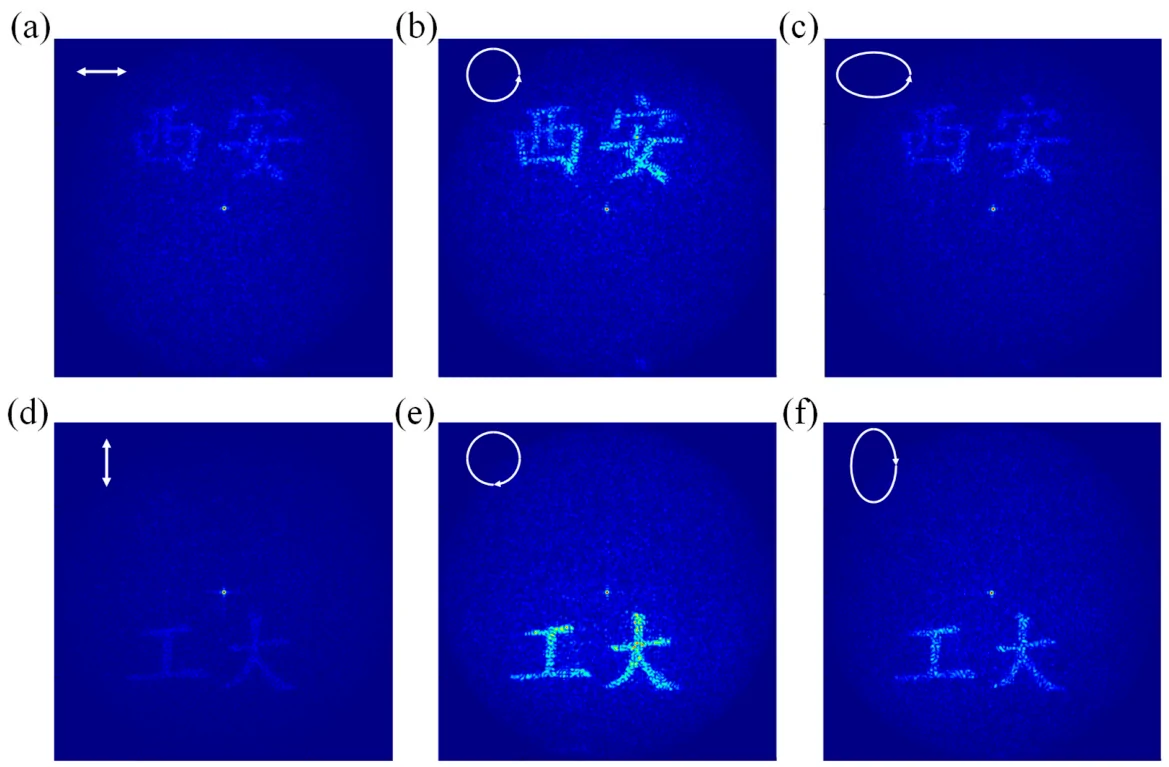
Simulation results of polarization multiplexing holography: (**a**,**d**) Linear polarization multiplexing; (**b**,**e**) Circular polarization multiplexing; (**c**,**f**) Elliptical polarization multiplexing. The Chinese characters in panels (**a**–**c**) and (**d**–**f**) read “Xi’an” (the name of a city in China) and “Gongda” (an abbreviation of *Gongye Daxue*, meaning “University of Technology”), respectively.

A meta-atom database was established from the periodic-unit-cell simulations described in Section 3.1. The selection criterion required the power transmittance to exceed 0.70 for illumination polarized along either principal axis of each unrotated nanobrick. The selected geometries and their corresponding phase responses were subsequently used to construct the holographic metasurface. After excluding invalid entries from the database, the nanobrick dimensions at each lattice position were selected by matching the required principal-axis phases, *φ_x_*(*x*,*y*) and *φ_y_*(*x*,*y*), with the corresponding phase responses of the candidate nanobricks, *P_x_*_,*k*_ and *P_y_*_,*k*_. Each phase difference was first reduced modulo 2*π* and then wrapped to the interval [−*π*,*π*]. The candidate geometry minimizing the combined phase error in Equation (14) was selected. Subsequently, anisotropic structures with appropriate dimensions were arranged on the SiO_2_ substrate with equal intervals (P = 1 μm) along both x and y directions, while applying corresponding rotation angles *θ*(*x*,*y*) to complete the device configuration. Figure 6 presents simulation results for three orthogonal polarization pairs: orthogonal linear polarization (Figure 6a,d), orthogonal circular polarization (Figure 6b,e), and orthogonal elliptical polarization (Figure 6c,f). All sub-figures clearly display the Chinese characters “Xi’an” and “Gongda”, maintaining excellent consistency with the original image.

(14) $$\varepsilon_{k}(x,y)=\sqrt{{\left\{{\mathrm{wrap}}_{\left[-\pi ,\pi \right]}[\varphi_{x}(x,y)-P_{x,k}]\right\}}^{2}+{\left\{{\mathrm{wrap}}_{\left[-\pi ,\pi \right]}[\varphi_{y}(x,y)-P_{y,k}]\right\}}^{2}}$$

Here, *k* denotes the candidate nanobrick index and wrap_[−*π*,*π*]_ denotes phase wrapping to the interval [−*π*,*π*].

The central bright spot in Figure 6 is associated with the zero-spatial-frequency component of the transmitted field. Under normal incidence, the on-axis far-field amplitude is proportional to the coherent sum of the transmitted fields across the metasurface aperture. A nonzero spatially averaged transmitted field therefore produces a central diffraction spot, whose angular width is determined by the illuminated aperture.

For circularly polarized illumination, this contribution can be examined through the complex transmission coefficients along the two principal axes of a nanobrick, $t_{u}=a_{u}e^{i\phi_{u}}$ and $t_{v}=a_{v}e^{i\phi_{v}}$. The co-polarized and cross-polarized transmission coefficients are

(15) $$t_{\mathrm{co}}=\frac{t_{u}+t_{v}}{2},\;\;t_{\mathrm{cross}}=\frac{t_{u}-t_{v}}{2}$$

The cross-polarized component additionally acquires the orientation-dependent geometric phase used for holographic encoding. The residual co-polarized intensity is governed by both the amplitude balance and phase retardation:

(16) $$|t_{\mathrm{co}}{|}^{2}=\frac{a_{u}^{2}+a_{v}^{2}+2a_{u}a_{v}\cos(\phi_{u}-\phi_{v})}{4}$$

Complete cancellation of this component occurs for equal principal-axis amplitudes and a phase difference of *π* modulo 2*π*. Deviations from these conditions leave a residual co-polarized field, whose nonzero spatial average contributes to the on-axis spot. Across the assembled hologram, spatial variations in transmission amplitude and phase-matching errors also modify the coherent on-axis field. This analysis connects the central spot to the realized complex transmission response of the metasurface. The polarization-filtering results in Section 3.4 further show that the central signal can be substantially suppressed while retaining the reconstructed images.

### 3.4. Polarization-Dependent Reconstruction and Suppression of the Zero-Order Spot

To quantify the dependence of image reconstruction on the polarization of the reading beam, the circular-polarization-multiplexed metasurface was further analyzed at a wavelength of 1550 nm. Two simulations were performed with identical device geometry and numerical settings, using equal-amplitude x- and y-polarized source components with relative phases of +90° and −90°, respectively. The corresponding incident polarization states are denoted by CP+ and CP−, with normalized Jones vectors ${\left(1,+i\right)}^{T}/\sqrt{2}$ and ${\left(1,-i\right)}^{T}/\sqrt{2}$. The exported complex vector far fields are denoted by E_+_ and E_−_. For the linear optical response considered here, intermediate incident polarization states were evaluated by coherent superposition of these two fields:

*E*(*β*) = cos*βE*_+_ + sin*βE*_−_ (17)

The mixing parameter *β* was varied from 0° to 90° in steps of 5°. The endpoints correspond to CP+ and CP− illumination, whereas *β* = 45° corresponds to x-polarized illumination. The orthogonality and equal incident powers of the two basis states ensure that the total incident power remains constant along this polarization path. These intermediate states were obtained by post-processing the two simulated complex fields, rather than by separate simulations at each *β*.

The reconstructed intensity was evaluated without an output polarization analyzer, using *I*(*β*) = |*E_x_*(*β*)|^2^ + |*E_y_*(*β*)|^2^ + |*E_z_*(*β*)|^2^. Two fixed rectangular regions of interest (ROIs) were defined in the far-field direction-cosine coordinates (*u_x_*,*u_y_*). The upper ROI containing “Xi’an” was bounded by −0.65 ≤ *u_x_* ≤ 0.65 and 0.10 ≤ *u_y_* ≤ 0.70; the lower ROI containing “Gongda” was bounded by −0.65 ≤ *u_x_* ≤ 0.65 and −0.70 ≤ *u_y_* ≤ −0.10. The ROIs were kept unchanged for all polarization states and excluded the central zero-order spot. The signal in each ROI was calculated with the solid-angle weighting appropriate to the hemispherical far-field projection:

(18) $$S_{j}(\beta )=\sum_{(u_{x},u_{y})\in \Omega_{j}}I(u_{x},u_{y};\beta )\frac{\Delta u_{x}\Delta u_{y}}{\sqrt{1-u_{x}^{2}-u_{y}^{2}}}$$

Here, *j* denotes the upper or lower ROI, and Δ*u_x_* and Δ*u_y_* are the sampling intervals. Both signals were normalized by a single reference value, defined as the maximum ROI signal over both channels and all sampled polarization states:

(19) $$J_{j}(\beta )=\frac{S_{j}(\beta )}{S_{\mathrm{ref}}},\;\;S_{\mathrm{ref}}=\max_{k,\beta^{\prime }}S_{k}(\beta^{\prime })$$

**Figure 7:**
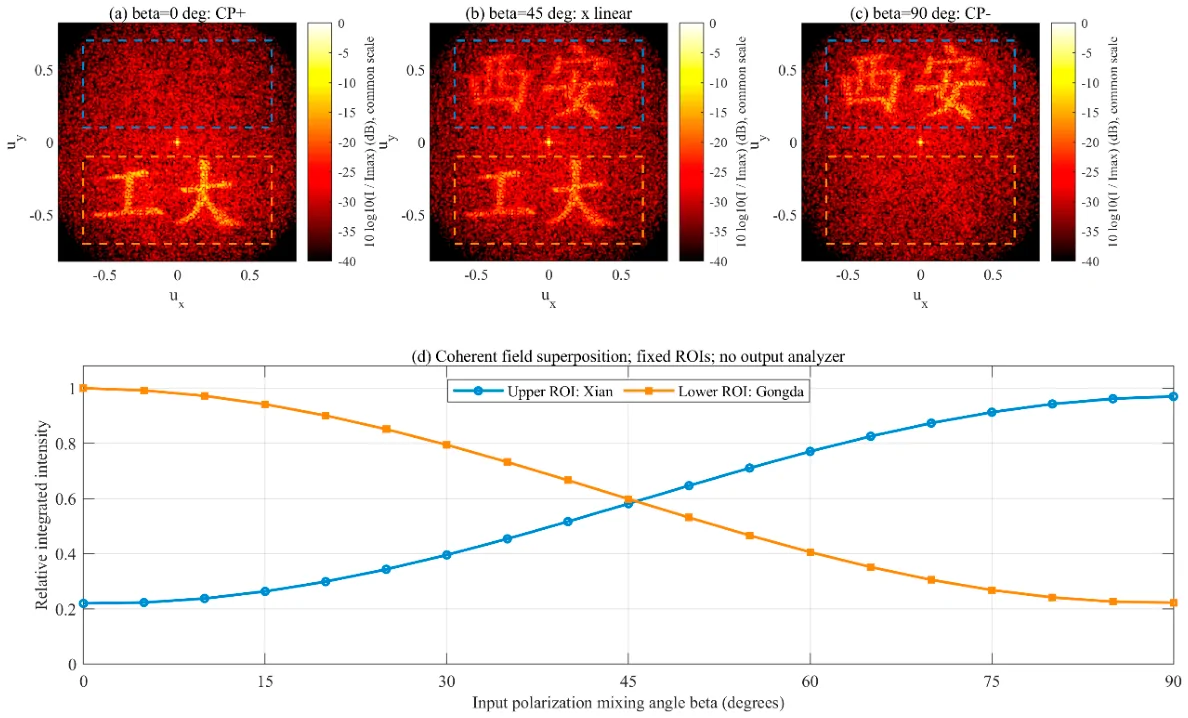
Polarization-dependent holographic reconstruction and relative intensity response. The Chinese characters in panels (**c**) and (**a**) read “Xi’an” (the name of a city in China) and “Gongda” (an abbreviation of *Gongye Daxue*, meaning “University of Technology”), respectively.

Figure 7a–c shows the reconstructed intensity distributions under CP+, x-polarized, and CP− illumination, respectively, and Figure 7d summarizes the polarization-dependent ROI signals. As β increases from 0° to 90°, the normalized signal in the upper “Xi’an” ROI increases from 0.220 to 0.970, while that in the lower “Gongda” ROI decreases from 1.000 to 0.223. At *β* = 45°, both images are reconstructed simultaneously, with normalized ROI signals of 0.582 and 0.599, respectively. These results demonstrate continuous control of the relative reconstructed intensities through the incident polarization.

To investigate whether polarization filtering can suppress the central zero-order spot, we further analyzed the polarization-resolved complex far fields at 1550 nm. An ideal collection and polarization-analysis model was adopted. The Cartesian field components were first converted into the local transverse components *E_θ_* and *E_ϕ_*. Each propagation direction was then mapped to a common transverse reference frame through a lossless meridional rotation, giving

(20) $$U=\left(\begin{array}{c} \cos\phi E_{\theta }-\sin\phi E_{\phi }\\ \sin\phi E_{\theta }+\cos\phi E_{\phi } \end{array}\right)$$

where *ϕ* is the azimuthal angle. This transformation preserves the intensity associated with each propagation direction. The model represents ideal collection and collimation and does not include finite collection aperture, polarization aberrations, or the angle-dependent transmission and retardance of practical optical components.

The central region was defined by $u_{x}^{2}+u_{y}^{2}\leq 0.03^{2}$, corresponding to a half-cone angle of approximately 1.72°. The image regions were identical to those used in the preceding intensity analysis. For a normalized transmitted Jones vector **q**, the signal after polarization analysis was calculated as

(21) $$P_{j}(q)=\sum_{\Omega_{j}}{\left|q^{†}U\right|}^{2}\frac{\Delta u_{x}\Delta u_{y}}{\sqrt{1-u_{x}^{2}-u_{y}^{2}}}$$

where *j* denotes the central or target-image region. The central residual fraction and image-signal retention were defined as

(22) $$L_{0}=\frac{P_{0,\mathrm{filtered}}}{P_{0,\mathrm{unfiltered}}},\;\;R_{\mathrm{img}}=\frac{P_{\mathrm{img},\mathrm{filtered}}}{P_{\mathrm{img},\mathrm{unfiltered}}}$$

respectively. The corresponding central-signal suppression was evaluated as −10log_10_*L*_0_.

**Figure 8:**
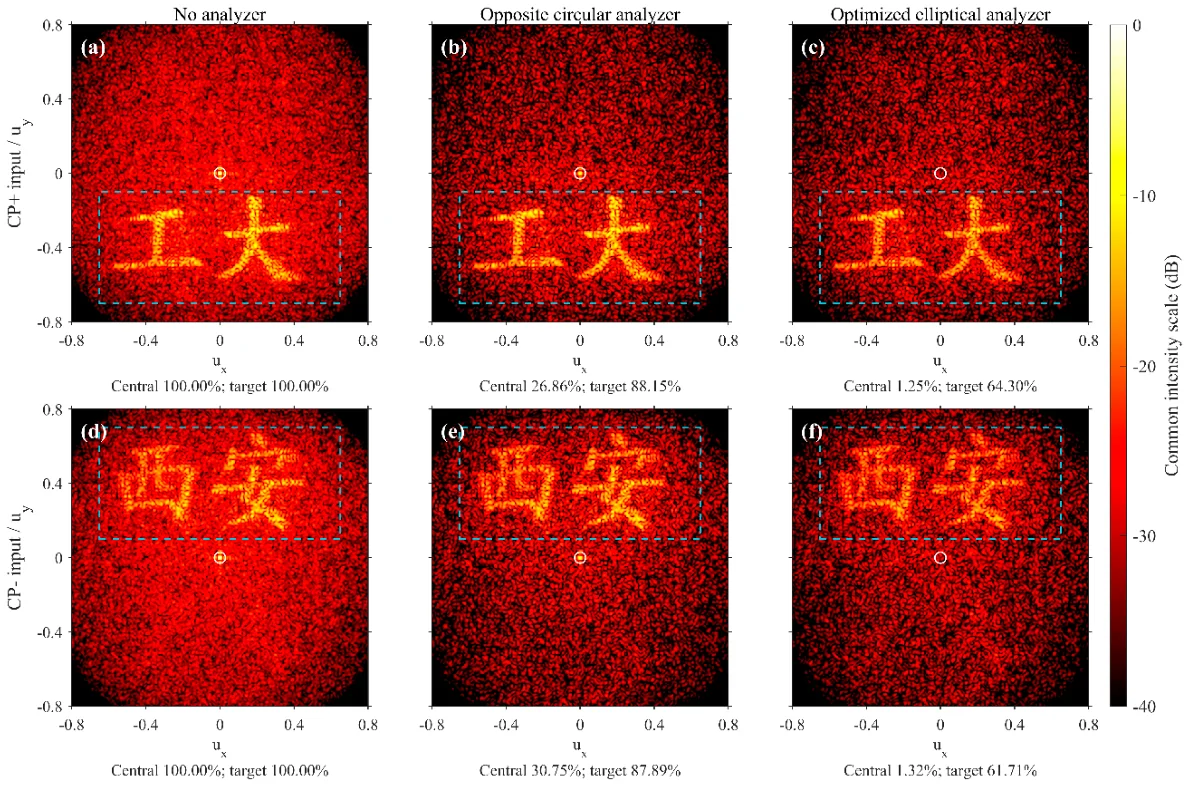
Polarization filtering of the reconstructed holographic images. (**a**) CP+ input without an analyzer; (**b**) CP+ input with an opposite circular analyzer; (**c**) CP+ input with an optimized elliptical analyzer; (**d**) CP− input without an analyzer; (**e**) CP− input with an opposite circular analyzer; (**f**) CP− input with an optimized elliptical analyzer. White circles and cyan dashed rectangles indicate the central and target-image regions, respectively. All panels share a common intensity scale in dB. The Chinese characters in panels (**d**–**f**) and (**a**–**c**) read “Xi’an” (the name of a city in China) and “Gongda” (an abbreviation of *Gongye Daxue*, meaning “University of Technology”), respectively.

Figure 8 compares the reconstructed intensity distributions under CP+ illumination in panels (a–c) and CP− illumination in panels (d–f). From left to right, the columns show the results without an analyzer, with an opposite-circular analyzer, and with an optimized elliptical analyzer. All panels use a common logarithmic intensity scale. The circles and dashed rectangles mark the central and target-image integration regions, respectively. The percentages below each panel indicate the central residual fraction and target-image signal retention relative to their respective unfiltered values. The opposite-circular analyzer selects the output handedness opposite to that of the incident beam. For the elliptical analyzer, the transmitted state was chosen as the eigenvector associated with the smallest eigenvalue of the central-region matrix

(23) $$C0=\sum \Omega_{0}UU^{†}\Delta \Omega$$

This choice minimizes the integrated central signal among spatially uniform ideal analyzers. The analyzer was optimized separately for the two incident polarization states and therefore requires different settings when the input polarization is switched.

For CP+ illumination, opposite-circular analysis retains 88.15% of the target-image signal while leaving 26.86% of the central signal. For CP− illumination, the corresponding values are 87.89% and 30.75%. The optimized elliptical analyzers reduce the central residual fractions to 1.25% and 1.32%, corresponding to suppression levels of 19.02 and 18.78 dB, while retaining 64.30% and 61.71% of the respective target-image signals.

The central and target regions cannot be described as two spatially uniform, strictly orthogonal polarization states. In the common transverse reference frame, the squared overlaps between their normalized dominant polarization eigenvectors are 0.314 and 0.346 for CP+ and CP− illumination, respectively. Furthermore, the central-region matrices have nonzero minimum eigenvalues, indicating that a single spatially uniform analyzer cannot completely extinguish the integrated central signal within this model. These results demonstrate a trade-off between central-spot suppression and target-image signal retention.

To examine polarization selectivity when the reconstructed images occupy the same spatial region, an additional metasurface hologram was designed with the “Xi’an” and “Gongda” targets positioned within the same off-axis reconstruction area. Two FDTD simulations were performed at 1550 nm under CP+ and CP− illumination with equal incident amplitudes. The resulting images demonstrate that spatial separation is not required for polarization-selective reconstruction.

**Figure 9:**
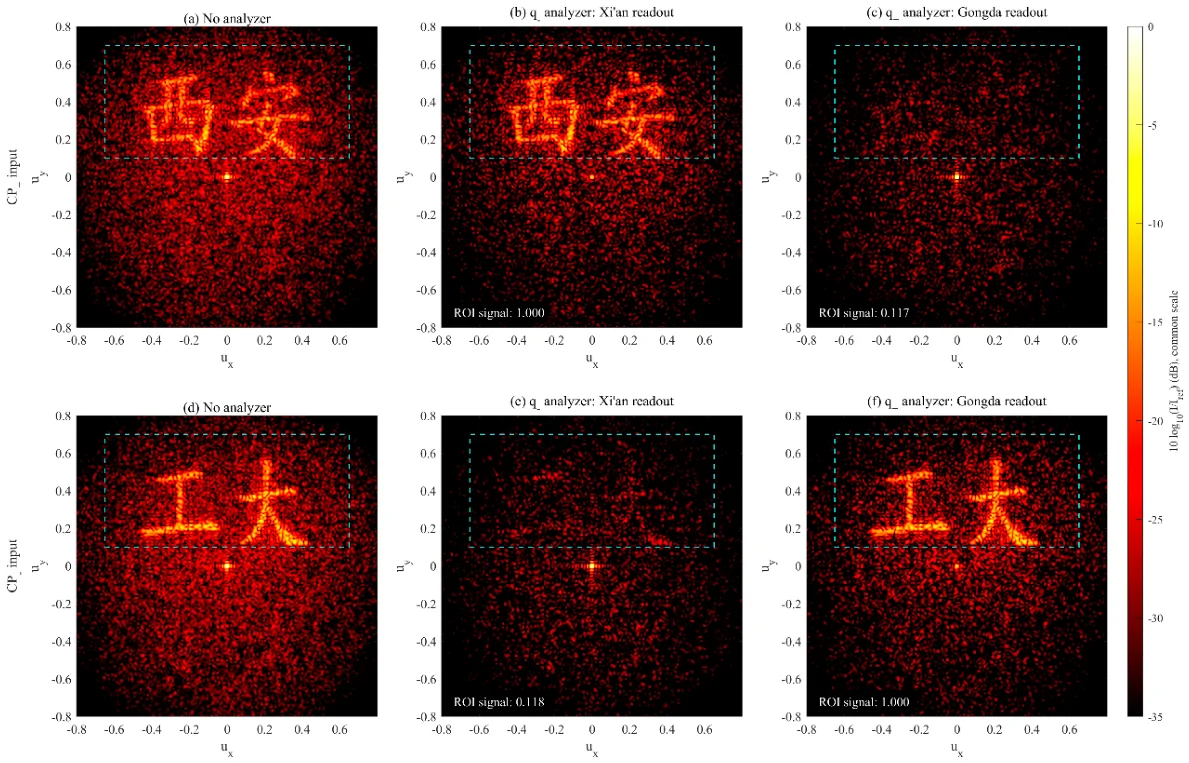
Polarization-selective reconstruction of spatially overlapping images. The Chinese characters in panels (**a**–**c**) and (**d**–**f**) read “Xi’an” (the name of a city in China) and “Gongda” (an abbreviation of *Gongye Daxue*, meaning “University of Technology”), respectively.

Figure 9 presents the reconstructed intensity distributions under CP+ illumination in panels (a–c) and CP− illumination in panels (d–f). The columns show the results without an analyzer, with a fixed CP− analyzer, and with a fixed CP+ analyzer, respectively. The analyzer vectors are defined as $q_{\pm }={\left(1,\pm i\right)}^{T}/\sqrt{2}$ in the common transverse reference frame introduced above. All six maps use a common logarithmic intensity scale. The dashed rectangles indicate the shared integration region, Ω: |*u_x_*| ≤ 0.65, 0.10 ≤ *u_y_* ≤ 0.70, which contains both reconstructed patterns while excluding the central zero-order spot.

The polarization-channel extinction ratio was evaluated by keeping the output analyzer and integration region fixed while switching the incident circular polarization. The integrated signal is defined as

(24) $$P_{s,t}=\iint_{\Omega }{\left|q_{s}^{†}U_{t}(u_{x},u_{y})\right|}^{2}\frac{du_{x},du_{y}}{\sqrt{1-u_{x}^{2}-u_{y}^{2}}}$$

where *s* denotes the analyzer state, *t* denotes the incident state, and **U***_t_* is the transverse field obtained using the preceding ideal collection model. Common factors relating the field integral to power cancel in the ratios. The extinction ratios of the two readout channels are

(25) $${\mathrm{ERXi}}^{\prime }\mathrm{an}=10\log_{10}\left(\frac{P_{-,+}}{P_{-,-}}\right),\;\;\mathrm{ERGongda}=10\log_{10}\left(\frac{P_{+,-}}{P_{+,+}}\right)$$

The calculated extinction ratios are 9.30 dB for the “Xi’an” channel and 9.33 dB for the “Gongda” channel. When normalized to the corresponding on-state signal, the residual signals under orthogonal illumination are 0.1176 and 0.1168, respectively. The ROI signal annotations in Figure 9 use this channel-specific normalization. These results demonstrate polarization-selective reconstruction within a shared spatial region, with finite residual leakage.

## 4. Fabrication and Experimental Characterization of Metasurfaces

**Figure 10:**
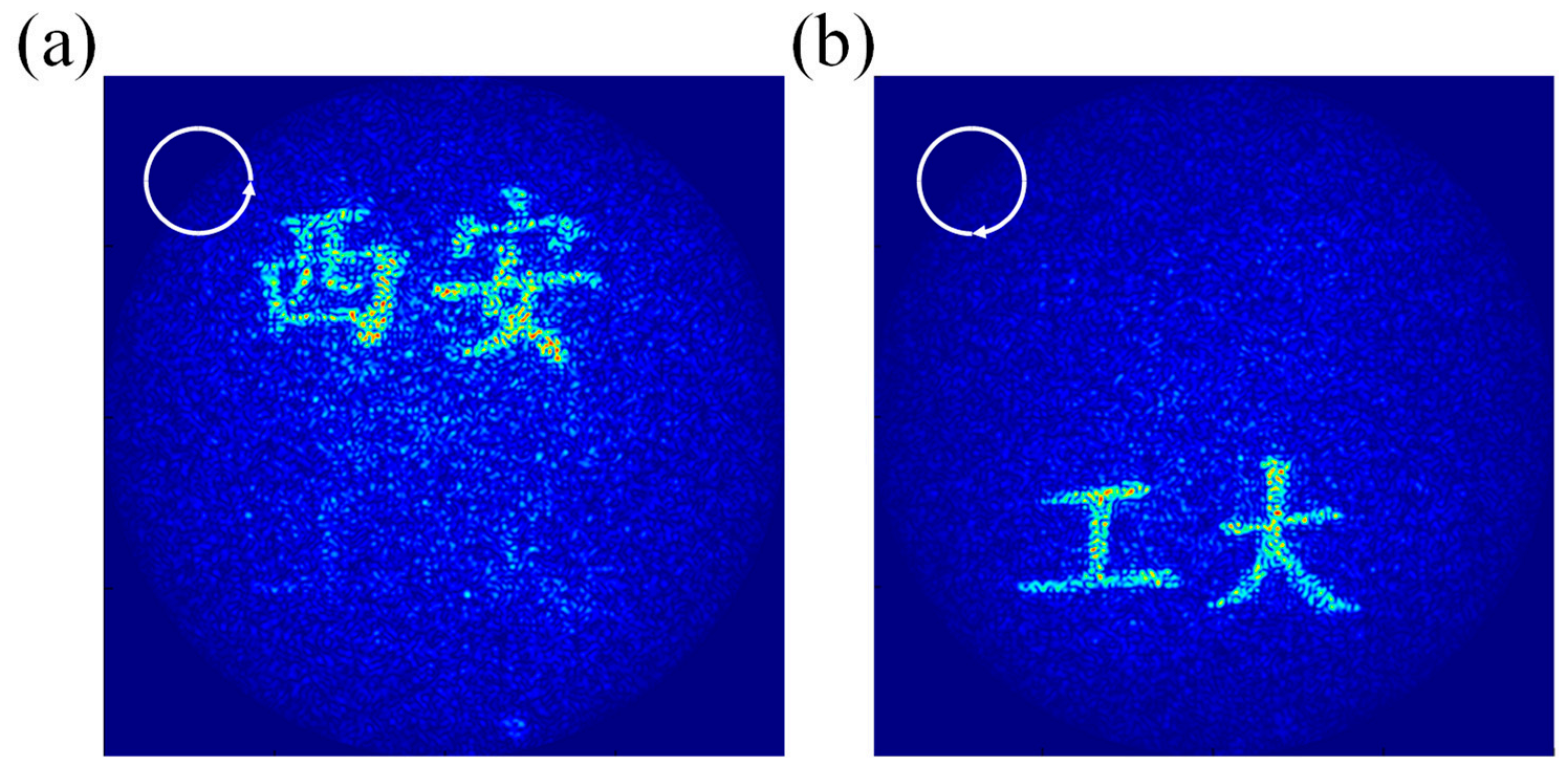
Simulation results after applying random errors: (**a**) Left-handed circularly polarized light imaging; (**b**) Right-handed circularly polarized light imaging. The Chinese characters in panels (**a**) and (**b**) read “Xi’an” (the name of a city in China) and “Gongda” (an abbreviation of *Gongye Daxue*, meaning “University of Technology”), respectively.

The designed circularly polarized multiplexed holographic metasurface was fabricated using electron beam lithography (EBL). During the fabrication process, dimensional errors inevitably occur, affecting the holographic imaging quality of the designed metasurface. Therefore, it is essential to analyze the impact of dimensional errors on the metasurface’s imaging performance prior to fabrication. Due to fabrication constraints, when processing features above 200 nm, dimensional errors not exceeding ±5% may be introduced. To account for this, random errors within ±5% were applied to the length, width, and height of the nanobricks in FDTD simulations. As shown in Figure 10, the simulation results demonstrate no significant degradation in image quality compared with Figure 6b.

**Figure 11:**
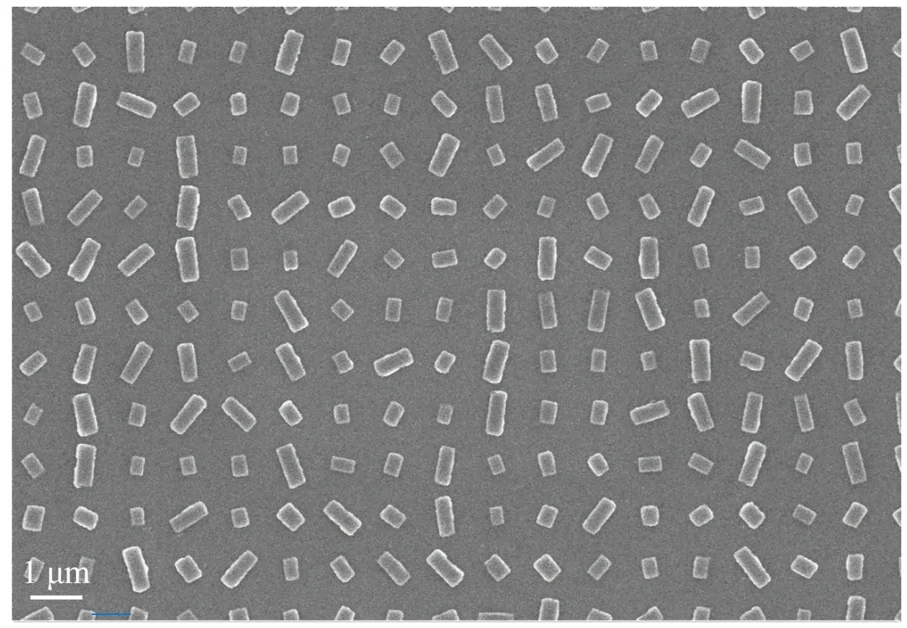
Morphological characterization of the metasurface structure.

The sample was fabricated using a combination of EBL and hard mask etching processes. The detailed fabrication steps are as follows: First, a 1 × 1 cm^2^ SiO_2_ substrate was cleaned using an organic cleaning station to remove surface contaminants and organic residues. After cleaning, a 1.2-μm-thick Si film was deposited on the substrate using electron beam evaporation. Subsequently, a 360-nm-thick ZEP photoresist layer was spin-coated as the top exposure layer. Electron beam lithography was then performed with a fixed beam current of 2 nA, selectively irradiating specific regions of the photoresist to create the desired pattern. During development, the exposed regions of the ZEP resist were removed to form the patterned openings. A chromium (Cr) metal film was subsequently deposited via electron beam evaporation, followed by a lift-off process to transfer the pattern to the metal layer. Finally, the metal layer served as a hard mask for etching the sample. After removing residual masks, the designed metasurface structure was obtained. Figure 11 shows SEM images of the partial structural morphology of the fabricated sample. The images demonstrate well-defined fabrication quality, indicating that the processed sample largely meets the design specifications.

**Figure 12:**
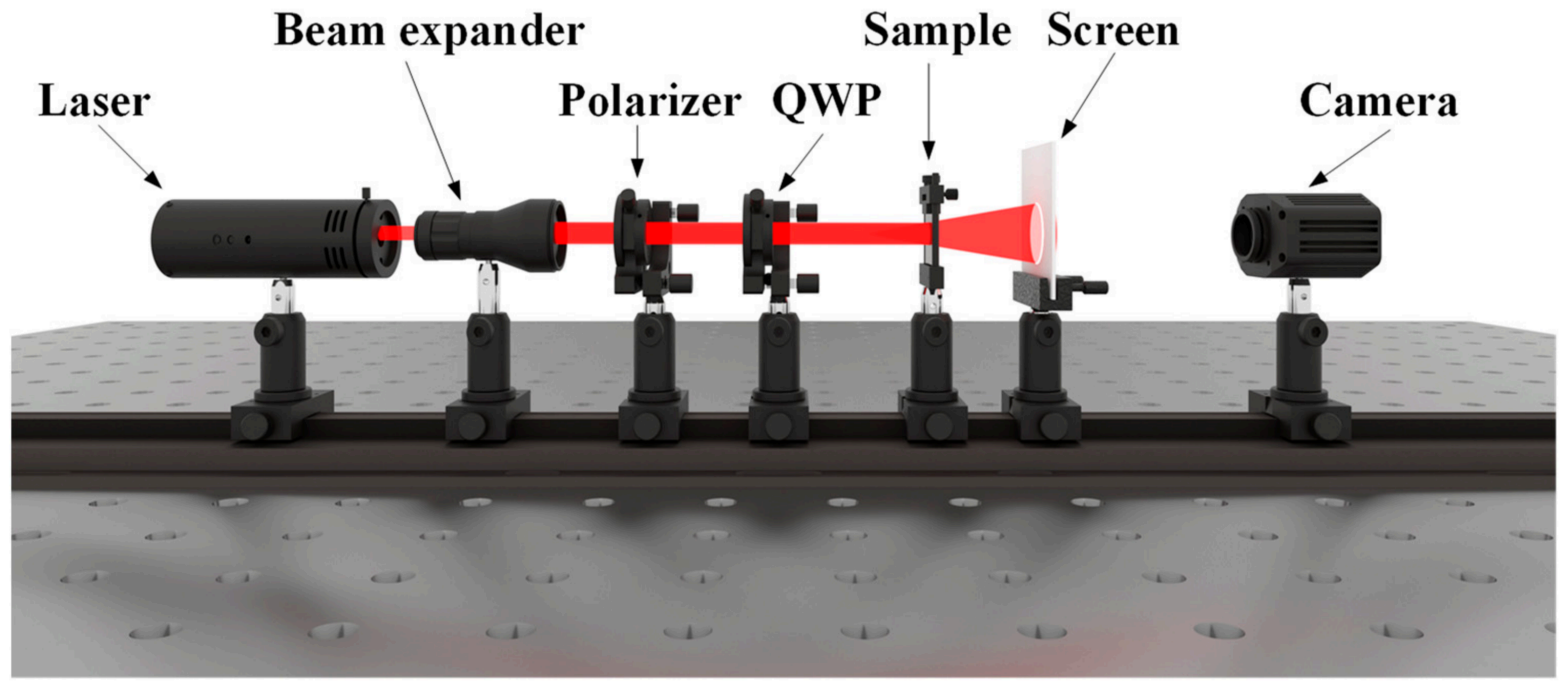
Experimental optical path diagram.

The experimental setup was constructed as shown in Figure 12. The emitted light from the source is expanded, collimated, and passed through the linear polarizer and quarter-wave plate to generate circularly polarized light. By adjusting the angular relationship between the fast axis of the quarter-wave plate and the polarization direction of the linear polarizer, left-handed and right-handed circularly polarized light can be selectively obtained. The polarized light interacts with the test sample and subsequently illuminates the receiving screen positioned at the image plane. The resulting optical patterns are captured by the CCD camera, thereby enabling acquisition of imaging results for both polarization states.

**Figure 13:**
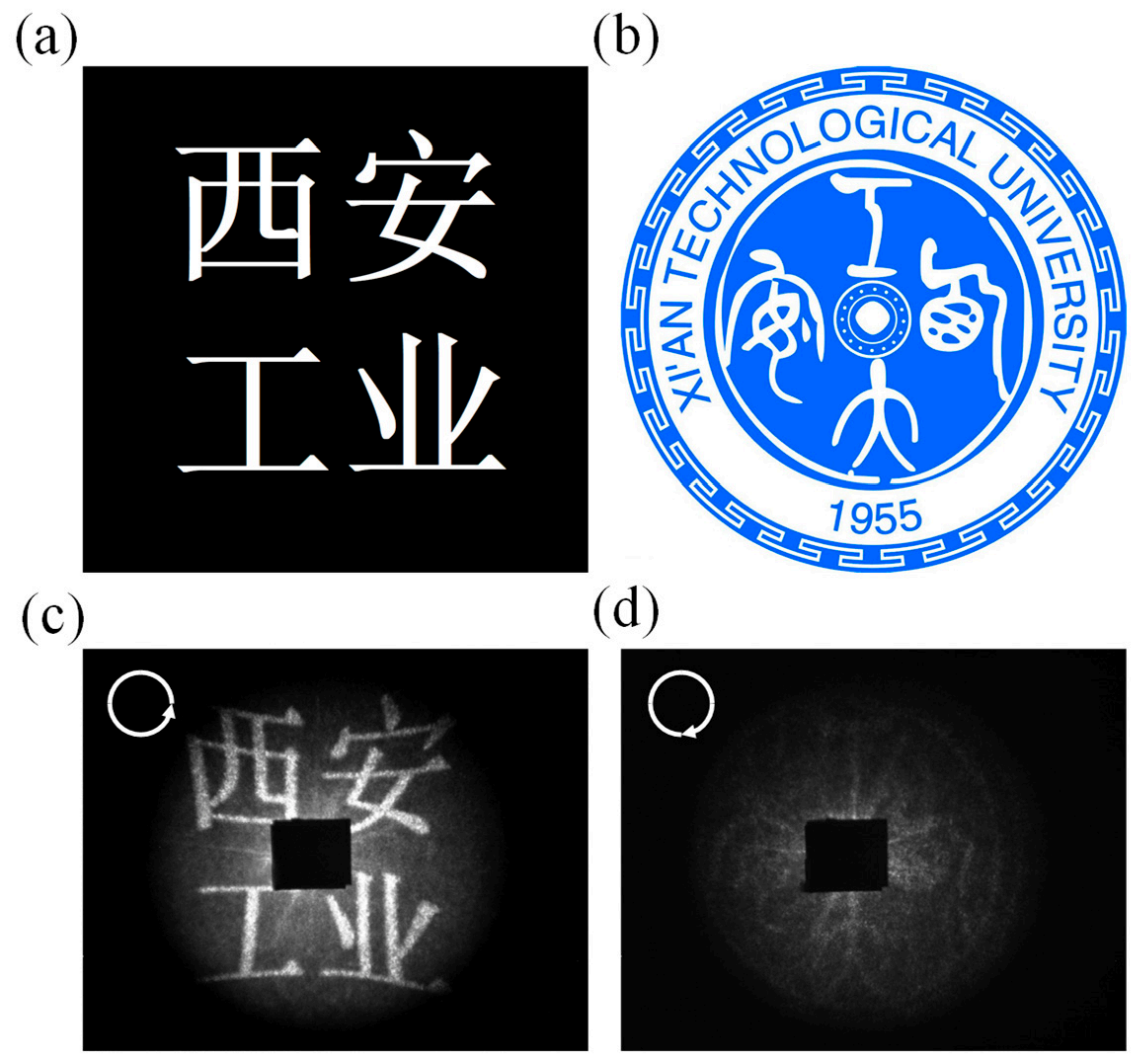
(**a**,**b**) Original image (**c**,**d**) Holographic image. The Chinese characters in panels (**a**) and (**c**) read “Xi’an” (the name of a city in China) and “Gongda” (an abbreviation of *Gongye Daxue*, meaning “University of Technology”), respectively.

Figure 13a,b show the target images, each represented by 1001 × 1001 pixels. Figure 13c,d present the corresponding experimental reconstructions under the two circular polarization states. The Chinese characters remain recognizable, with uneven stroke brightness and local contour deformation, whereas the university emblem exhibits a blurred overall outline and reduced visibility of its fine features. During image acquisition, a physical stop was used to block the central zero-order light, producing the black square visible in both reconstructed images. The camera was positioned facing the receiving screen.

The degradation outside the blocked region is consistent with imperfect reconstruction of the intended optical wavefront. In the design procedure, the target phase at each position is approximated using the available nanobrick geometries, introducing phase-matching errors. The selected nanobricks also exhibit variations in transmission amplitude. These phase and amplitude deviations modify the coherent interference of the transmitted fields, redistributing optical energy between the intended image features and the background. Consequently, the reconstructed images can exhibit nonuniform brightness, irregular stroke boundaries, and reduced feature contrast. Differences between the fabricated structures and the design, together with variations in the illumination across the sample, can further modify the transmitted wavefront.

The emblem contains more closely spaced lines and smaller features than the character pattern. Resolving these features requires sufficient optical resolution and accurate reproduction of their interference pattern, making their visibility more sensitive to wavefront errors and background intensity. This provides a physical explanation for the stronger loss of detail in the emblem reconstruction. The tolerance analysis in Figure 10 demonstrates robustness to the specified ±5% random dimensional perturbations, while the experimental images reflect the combined effects of phase matching, amplitude variations, fabrication, and illumination.

## 5. Conclusions

A silicon-nanobrick metasurface for dual-channel polarization-multiplexed holography at 1.55 μm was developed using combined propagation-phase and geometric-phase control. The metasurface employs a 1 μm lattice period and a nanobrick height of 1.2 μm. Target phase profiles obtained using a Fresnel-based GS algorithm were mapped to nanobrick dimensions and orientations through Jones-matrix design relations and wrapped phase-error minimization. Numerical simulations demonstrated reconstruction for orthogonal linear, circular, and elliptical polarization pairs. The circular-polarization metasurface was fabricated using electron beam lithography and hard-mask etching. Under the two orthogonal circular polarization states, the experimental far-field reconstructions produced the Chinese-character image and the overall envelope of the university emblem. The Chinese characters remained recognizable, while the finer features of the emblem exhibited reduced visibility. These experimental results demonstrate polarization-selective switching between two encoded images.

Analysis of the complex far fields quantified the dependence of the reconstructed intensities on the incident polarization and showed simultaneous reconstruction of both images under x-polarized illumination. Under the ideal collection and analyzer model, separately optimized elliptical analyzers suppressed the central signal by 19.02 and 18.78 dB for CP+ and CP− illumination, respectively, while retaining 64.30% and 61.71% of the corresponding target-image signals. An additional metasurface design placed both images within the same reconstruction region and achieved polarization-channel extinction ratios of 9.30 dB for the “Xi’an” channel and 9.33 dB for the “Gongda” channel under fixed circular-analyzer conditions. These results connect the design and experimental implementation of the metasurface with quantitative analysis of polarization-dependent holographic readout, supporting applications in polarization-based information encoding.

## Data Availability

The original contributions presented in this study are included in the article. Further inquiries can be directed to the corresponding author.
